# Migrant Sexual Health Help-Seeking and Experiences of Stigmatization and Discrimination in Perth, Western Australia: Exploring Barriers and Enablers

**DOI:** 10.3390/ijerph13050485

**Published:** 2016-05-11

**Authors:** Josephine Agu, Roanna Lobo, Gemma Crawford, Bethwyn Chigwada

**Affiliations:** 1 School of Public Health, Curtin University, Kent Street, Bentley, Western Australia 6102, Australia; 2 Collaboration for Evidence, Research and Impact in Public Health, School of Public Health, Curtin University, Kent Street, Bentley, Western Australia 6102, Australia; roanna.lobo@curtin.edu.au (R.L.); g.crawford@curtin.edu.au (G.C.); 3 HepatitisWA (Inc.), 134 Aberdeen Street, Northbridge, Western Australia 6003, Australia; BCDO@hepatitiswa.com.au

**Keywords:** sexual health, migrants, HIV, health determinants, stigma, discrimination

## Abstract

Increasing HIV notifications amongst migrant and mobile populations to Australia is a significant public health issue. Generalizations about migrant health needs and delayed or deterred help-seeking behaviors can result from disregarding the variation between and within cultures including factors, such as drivers for migration and country of birth. This study explored barriers and enablers to accessing sexual health services, including experiences of stigma and discrimination, within a purposive sample of sub-Saharan African, Southeast Asian, and East Asian migrants. A qualitative design was employed using key informant interviews and focus group discussions. A total of 45 people with ages ranging from 18 to 50 years, participated in focus group discussions. Common barriers and enablers to help seeking behaviors were sociocultural and religious influence, financial constraints, and knowledge dissemination to reduce stigma. Additionally, common experiences of stigma and discrimination were related to employment and the social and self-isolation of people living with HIV. Overcoming barriers to accessing sexual health services, imparting sexual health knowledge, recognizing variations within cultures, and a reduction in stigma and discrimination will simultaneously accelerate help-seeking and result in better sexual health outcomes in migrant populations.

## 1. Introduction

Infections which were previously geographically confined are now universal public health concerns as a result of globalization [1]. Emerging and re-emerging infectious diseases, as well as antimicrobial drug resistant strains of infections, have substantially threatened global health [2]. The HIV/AIDS pandemic and Ebola outbreaks are examples of rapid global dissemination of infectious diseases that have affected low, middle, and high income counties [2].

In the last ten years, Australia has reported an increase in the number of HIV notifications particularly amongst individuals travelling in and out of high HIV prevalence regions [3]. This increase has been attributed in part to the growth in migration and mobility rates [4,5,6]. *The Seventh National HIV Strategy 2014–2017* in Australia identifies migrants and other mobile populations as priority populations citing a range of challenges for prevention, treatment, care, and support, including help-seeking, access, culture, gender, stigma, and discrimination [7]. Australia is well placed to support other countries committed to limiting HIV within their borders. The Australian public health response to HIV has been recognised globally and has been referred to as “a public health milestone of global importance” [8,9]. Key features of the response were the bottom-up approach that prioritized concerns and facilitated the mobilization of affected communities and the development of partnerships between national and state and territory governments, clinicians, researchers, and other HIV-related organizations [8,9]. These collaborations provided a framework to establish policies that included evidence-informed research, care, prevention, and treatment [8]. The sustained outcomes of Australia’s timely response include an epidemic that is largely concentrated in specific vulnerable groups, with low HIV rates established in the general population (<1%) relative to other high-income countries, as well as a wealth of practical knowledge and experience [3,8,9].

However, despite these successes and the provision of comprehensive sexual health services, in 2008, the Australian national surveillance data revealed a 34% increase in the number of new HIV diagnoses among people from high HIV prevalence countries currently residing in Australia [10,11]. HIV diagnoses in the Australian heterosexual population have noticeably increased primarily in migrants from sub-Saharan Africa (SSA), Southeast Asia (SEA), and the Caribbean [3]. Australian-based research has shown that difficulties persist in ascertaining the extent to which existing facilities and services are meeting the sexual health needs of specific groups within the epidemic, including eligibility and delayed access for treatment [12,13].

Migrant groups are often conceptualized as homogenous, leading to less targeted approaches when planning and implementing support services for mobile and migrant populations [14,15]. Specific migrant needs are not always considered in service delivery which often ignores the spatial and temporal variation of migrants [16]. There are few culturally- and linguistically-diverse (CaLD)-specific services and a fragmented system of service delivery, reducing the likelihood of appropriate service access or referral [17]. Disregarding within and between cultural variations can lead to misguided generalizations about migrant health needs [16] and lower utilization rates of sexual health services among CaLD groups [18].

In 1963, Goffman defined stigma as ”an attribute that is significantly discrediting which, in the eyes of society, serves to reduce the person who possesses it” [19]. A stigmatized individual is referred to as someone who possesses “an undesirable difference” and the social, cultural, and moral process based around this perceived difference is referred to as stigmatization [19,20,21]. Negative experiences of stigmatization and discrimination can have adverse effects on migrant sexual health, quality of life, self-esteem, and sense of belonging [22] and have previously been identified as barriers to help-seeking and correlated with risky behaviours [12,23,24], Migrants may experience stigma and discrimination in terms of accessing healthcare services, employment, accommodation, education and social exclusion, all of which have been found to increase the likelihood of delayed or deterred help-seeking [11,25].

The availability of sexual health services is considered a human right, but there are globally recognised limitations to accessing these services [18]. Some previously-identified barriers to sexual health help-seeking include financial constraints, stigma, language barriers, and fear of deportation [26]. Research into migrant sexual and reproductive health has explored barriers and enablers to accessing services from a broad perspective, utilizing diverse “migrant” definitions, smaller sample sizes, and considering a wide range of cultures making comparisons more difficult. Additionally, research on sexual health needs in minority ethnic communities remains limited with very few studies exploring the impact of stigma and discrimination on sexual health within the migration experience [27].

In order to identify more specific needs and improve utilization of services, we sought to develop an understanding of these barriers alongside social, economic, cultural, and physical enablers to accessing sexual health services in migrants primarily from the sub-Saharan African (SSA) and Southeast Asian (SEA) regions consistent with changes in the HIV epidemic [28].

## 2. Materials and Methods

The consolidated criteria for reporting qualitative research (COREQ) [29] for explicit and comprehensive reporting of qualitative studies was used to guide the study design and report on its findings. Components of the three COREQ domains were addressed: Domain 1—Research team reflexivity, Domain 2—Study design, and Domain 3—Analysis and findings. The main methods used in this study were key informant interviews and focus groups discussions (FGDs) [29].

### 2.1. Research Team and Reflexivity

The research team comprised four members including two university-based academics, a public health research student, and a not-for-profit organization manager working with migrant communities, all with previous experience in conducting research. Key areas of expertise represented by the team were qualitative research methods, evaluation, and experience in working with minority, vulnerable, and multicultural groups. All team members had interests in the research topic as well as sexual health-related backgrounds. Some had previously participated in community development, advocacy, health promotion, and hospital-based sexual health service delivery. In some cases, where members of the team had existing ties with some of the research participants, a different researcher was assigned to be the primary facilitator of the FGDs.

### 2.2. Methodology

A constructivist paradigm was chosen to facilitate the comprehension and explanation of the multilayered social realities and lived experiences of participants [30]. In constructivism, knowledge and truth is perceived as a phenomenon that is actively constructed by individuals and guides their actions, notions, and feelings [31].

### 2.3. Study Setting

The study was conducted in the Perth metropolitan area in Western Australia (WA) and data were collected at locations that were convenient for participants (university, multicultural centre) [29]. In 2011, there were approximately 2.35 million WA residents (10% of Australia’s population) [32]. Among all the Australian states and territories, WA has the highest proportion of its population born overseas (27%) [32,33]. Over 200 countries, 100 religions and 170 languages are represented in WA alone, making cultural and linguistic diversity a key feature [34,35]. More than 75% of WA’s CaLD populations reside in the Perth metropolitan area [36].

In WA, there are a number of general, sexual, and reproductive health services available at general practices and metropolitan and statewide clinics. There are only are a few sexual health-specific clinics, mainly attached to hospitals. Sexual health helplines are also available. Sexual health services are free to Australian citizens and permanent residents, however temporary residents and migrants are ineligible for subsidized Medicare, as well as antiretroviral therapies (ARTs) after diagnosis with HIV [37,38].

### 2.4. Operational Definitions

The term “migrants” in this study refers to individuals who are currently living in Australia but were born overseas in one of the target group regions (sub-Saharan Africa and Southeast Asia) and who entered Australia on a student, humanitarian, family, or 457 temporary work visa. Some study participants still held the visas used to enter Australia, some had transitioned to permanent residency/citizenship, and others were issued permanent residency upon arrival in Australia. sub-Saharan African migrants were those born in any of the countries within sub-Saharan Africa and Southeast Asian migrants were those born in any of the countries classified according to the Australian Standard Classification of Countries for Social Statistics including: Burma (Myanmar), Vietnam, Brunei, Malaysia, Cambodia, Indonesia, Laos, Philippines, Singapore and Thailand.

### 2.5. Participant Eligibility and Recruitment

Eligible participants were adult males or females aged 18 years or more, born in sub-Saharan Africa or Southeast Asia [39], and who had lived in Perth for one year or more. This time period was considered long enough to have had significant migrant experience and a general/basic understanding of the Australian healthcare system. Recruitment occurred primarily through purposive and snowball sampling [29]. These were considered effective sampling methods because the study targeted a specific group and not the general migrant populace. Selection was made purposefully for information that only the target group and their affiliates could provide and that could not have been obtained from other groups [40,41]. Interested and eligible participants received information sheets prior to the FGDs and provided signed consent forms before study commencement.

Most migrants were recruited through flyers distributed face-to-face or through email. The settings from which some migrant participants were recruited included whole English language student classes. The classes were purposefully selected based on moderate to high English language proficiency and high numbers of students from sub-Saharan Africa or Southeast Asia. Student class members from countries outside the target group were permitted to participate in the focus groups; however their data were excluded. Nine participants for the key informant interviews were recruited by emails sent to a community development and advocacy team at a sexual health council and face-to-face meeting with a sexual health reference group. Both groups were selected for their extensive experience in sexual health communication for a range of priority target groups. Postgraduate qualitative research students, including students from multicultural backgrounds, were also recruited face-to-face to pilot test focus group activities. There was no participant attrition in the study [29]. All focus group participants were entered into a prize draw for gift vouchers and an iPad as an acknowledgement of their contribution.

### 2.6. Data Collection

Key informant interviews, pilot testing, and focus group discussions were all conducted in English.

#### 2.6.1. Key Informant Interviews

Key informants comprised

- A health promotion team leader at the local non-government AIDS organization with experience in health promotion in the areas of sexual health and blood borne viruses;
- Three health promotion officers working at the local non-government AIDS organization with experience working with at risk groups such as CaLD, young people, and Australian Aboriginal and Torres Strait Islander communities;
- One program coordinator at the resource centre for migrants with experience in working with CaLD communities and assisting them with settlement in Western Australia; and
- A community consultation group made up of multicultural members from a range of Sub-Saharan African and Southeast Asian countries who had migrated to and settled in Australia.

Each key informant interview lasted approximately 30 min. Interviewees worked with the researchers as part of a preliminary step to review, modify and establish an appropriate semi-structured guide for the FGDs based on their knowledge of the target groups. Open-ended questions and non-directive probes were then finalized relative to the study objectives to obtain full and unbiased responses during the FGDs. Sample interview questions included but were not limited to those shown in Table 1 under all seven domains covered.

#### 2.6.2. Pilot Testing

A pilot test of the focus group was conducted with a group of 13 postgraduate qualitative research students and lasted for approximately 40 min. The class selected for the pilot test comprised predominantly international students, 11 of whom were from a CaLD background. The pilot test was administered to trial the focus group proceedings, *i.e.*, content, flow, timing, sensitivity, and language of the questions and activities after which adjustments were made before conducting FGDs with the study participants. The discussion was audio recorded and field notes were taken for reference purposes but were not transcribed for analysis.

#### 2.6.3. Focus Group Discussions (FGDs)

Qualitative FGDs were chosen to elicit the underlying reasons behind certain behaviours and allowed participants to express themselves based on their experiences and understandings [42]. These methods have been found to be beneficial in highlighting future areas of inquiry and elucidating existent cross-cultural differences that are often missed due to cultural misconceptions [43]. The synergistic effects from discussing and contributing ideas are key features of focus groups that cannot be achieved during individual interviews [44,45].

Focus groups have proven efficient in working with diverse cultural groups as they foster group interaction and permit for divergence or consensus on issues and encourage participation from individuals who may otherwise be reluctant to be individually interviewed [43]. Sample size was determined by saturation, *i.e.*, when no new themes or ideas emerged [29]. There were 45 focus group participants in this study. The aim was to obtain a cross-section of viewpoints from a purposive cohort, only transferable within the target groups (SSA and SEA migrants) and not across the migrant population at large [43]. Five focus groups were held as evidence suggests that this number is sufficient to achieve some level of data saturation [46,47,48]. Focus groups contained five to 12 participants, an adequate number to generate discussion, but not too large that it impeded the contribution of other reticent members [49]. The duration of each focus group was approximately 90 min [29]. Focus groups were facilitated by a member of the research team as well as a co-facilitator who recorded field notes and observed the discussion to avoid missing relevant non-verbal cues [44].

### 2.7. Qualitative Data Analysis

Audio recordings were transcribed verbatim in English and transcripts were verified by replaying recordings whilst reading through the text and making corrections where necessary to ensure correlation. Data were analysed by two members of the research team using a modified grounded theory approach which included an inductive process to identify common themes, but not the deductive element of theoretical sampling or model building [50]. The transcripts were first read through twice as a complete set of data in order to establish a broader understanding of the emerging and common themes in the participants’ dialogue. Individual transcripts were then analysed thematically. Thematic analysis involves breaking down data into smaller units, typically sentences, or occasionally paragraphs, that convey discrete information, then assigning meaningful labels (or codes), which represent the data [51]. The codes were revisited during analysis of each transcript and adjusted if necessary to eliminate ambiguity and overlap and to verify accurate representation of the whole dataset. Following this initial coding, similar data were grouped together in categories. Eventually, consistent patterns (or themes) could be identified in the dataset.

### 2.8. Ethical Considerations

The Curtin University Human Research Ethics Committee approved this study (approval number RDHS-96-15). The research team members have all worked in sexual health and with vulnerable populations previously, and had undertaken research integrity and cultural competence training. In order to ensure that the methodology and instruments used were culturally appropriate and safe, key informants formed an advisory group that was engaged as part of the formative evaluation process. Questions included in the discussions were workshopped prior to implementation to reduce the likelihood of embarrassment or discomfort. During focus group discussions, two trained facilitators explained the rationale for the study and the risks and benefits of participation. Facilitators also ensured that unfamiliar terminologies were defined and understood. Participants were given information sheets, which used lay terms to ensure comprehension, they provided written consent, and were given the option to withdraw from the research at any point in time. To address the difficulty often associated with discussing sensitive sexual health-related topics, participants were not required/obliged to share personal experiences unless they were comfortable doing so. They were able to communicate in the third person often using the experiences of close relatives, friends or hypothetical characters to express their views. Scenarios and icebreaker activities were used to support the development of rapport, trust, and the creation of a safe space to share ideas. In addition to this, focus group sessions were conducted at multiple locations that were convenient and familiar to participants to ease discussion in a public setting. An adverse response protocol was adopted in the event that participants experienced any emotional discomfort during the proceedings and referrals to a range of services were made available to participants. Participants were offered refreshments to acknowledge the time taken to participate. Data was only made accessible to the researchers and stored securely in a database. After transcription, audio recordings were destroyed and discarded. Confidentiality was assured as anonymity was maintained during data analysis and only gender and country of origin were used to demonstrate the diversity of responses represented by example quotes in reporting.

### 2.9. Socio-Demographic Characteristics of Participants

The total number of focus group participants was 45, comprising a larger number of female than male participants (*n* = 28 and *n* = 17, respectively). The majority identified as Christian (*n* = 27), participants’ ages ranged from 18 to 50 years with the predominant age group being 18–28 years (*n* = 24), just over half had completed a University bachelor degree or higher (*n* = 24) and a larger proportion had lived in Australia for between one to five years (*n* = 34) while the rest had been residents for more than five years (Table 2). Other participants outside the target group were present in two of the FGDs, they were from East Asia and other regions. Responses from the East Asian participants were included in the analysis and reporting as a result of their close proximity to Southeast Asia, while responses from participants from other regions were excluded. The twelve countries represented from sub-Saharan Africa were Burundi (1), Congo (1), Ethiopia (2), Ghana (1), Kenya (3), Liberia (1), Malawi (1), Namibia (1), Nigeria (4), Sudan (2), The Gambia (1), and Zambia (3). Five Southeast Asian countries were represented with the highest number of participants from Vietnam (6) followed by Malaysia (2), Philippines (1), Indonesia (1), and Brunei (1).

East Asian participants outside the target group were from Japan (2) South Korea (2), China (2), Hong Kong (1), and Taiwan (1). Participants from other regions also outside the target group were from Afghanistan (2), Iran (2), and Serbia (1).

### 2.10. Study Limitations

This study represents a snapshot of views on sexual health help-seeking from migrants from SSA and SEA/EA, reflecting a particular time and place. Whole class recruitment limited our ability to restrict participation to solely the target group countries. The presence of non-Asian participants in the SEA/EA focus groups may have resulted in reluctance by SEA/EA migrants to contribute adequately. This limited the number of themes from the SEA/EA groups compared to the sub-Saharan African group, which were comprised entirely of migrants from sub-Saharan Africa. There may be a tendency for extroverts to be over represented in focus group discussions [52]. Participants’ contributions may have also been influenced by social desirability bias, where some people may agree with answers that are socially desirable and acceptable in a group setting rather than giving responses that are reflective of their true feelings [53].

Time constraints did not enable for further recruitment into the study to explore SEA/EA experiences in more detail. There were also language challenges making it difficult for some participants to articulate and contribute to the conversation by agreeing or disagreeing. The involvement of interpreters is suggested to alleviate this challenge. Finally, participants were predominantly students who may often be less economically stable than working adult migrants that are able to access services without financial constraints. Higher levels of education may also not be reflective of the wider migrant population.

## 3. Results

**Figure 1:**
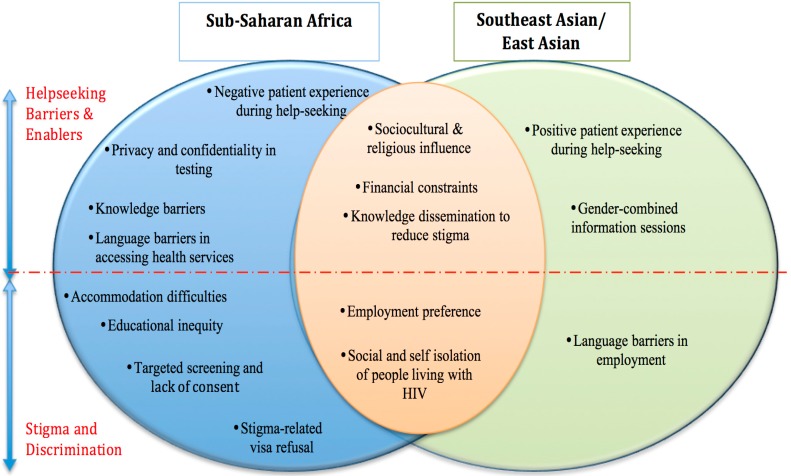
Venn diagram showing commonalities and differences across groups.

This study reports findings from the focus group discussions. For both sub-Saharan African and Southeast Asian/East Asian groups, there were five common themes including sociocultural and religious influences, financial constraints, knowledge dissemination to reduce stigma, employment preference, and social and self-isolation of people living with HIV. There were eight themes specific to the sub-Saharan African cohort, three themes from the Southeast Asian /East Asian focus groups, and five themes common to both groups (Figure 1). There is a distinct division of themes across both target groups into those pertinent to help-seeking barriers and enablers and others that are related to participants’ experiences of stigma and discrimination (Figure 1).

### 3.1. Negative Patient Experience during Help-Seeking

Several sub-Saharan African participants construed past help-seeking experiences in Australia as less than satisfactory, often referring to the process as tedious particularly for non-Medicare cardholders. Common sources of help cited by female participants in this cohort, in order of frequency, were general practitioners (GPs), tertiary hospitals, and sexual health clinics while male participants were unfamiliar with these services or their locations, but the majority also cited GPs. GPs were notably the first points of contact for Southeast Asian/East Asian and sub-Saharan African participants with a number of Southeast Asian /East Asian participants saying they would request for more specific referrals from the GPs. Delayed service delivery and an unclear understanding of the health system were contributors to substandard patient experiences which sometimes left participants questioning the effectiveness and fairness of the health system.

*“…the GP gave me a referral and then after the referral it took almost a month before we had to go to the hospital for a test, so I don't know if it is like that for everybody”*.

(Female from Liberia)

Some Southeast Asian/East Asian participants expressed a preference for family doctors over unfamiliar GPs however there were no complaints on services rendered, the majority experienced equal treatment.

*“…if we go to the hospital or clinic, everyone is equal because last time I went to the clinic, GP, I still had to wait like everyone else and we all followed the same steps to get seen”*.

(Female from Hong Kong)

### 3.2. Sociocultural Influences on Sexual Health

Cultural influence was expressed within the context of the uneasiness in communicating about sexual health due to the negative connotations attached to sexual health in both the sub-Saharan African and Southeast Asian/East Asian focus groups. This meant that exposure to these topics occurred primarily after migration to Australia. Parents were often indirect in communication sometimes using frightening stories of teenage pregnancy or STIs as strategies to prevent sexual activity and seldom suggesting safe sex as an alternative. Parents also apportioned minimum significance to sexual health compared to areas like education.

*“In my country it’s a taboo because of my religion (Muslim)... we are being told that it is a taboo and we do not talk about these things”*.

(Male from Indonesia)

*“For us, the sexual health thing was a taboo to talk about in the home…but then when we came here, I was so exposed to all these sexual orientations, sexual health is not talked about a lot so we didn't know much, all they told us was stay away from boys and don't touch boys”*.

(Female from Congo)

*“Parents don't talk to their kids about sexual health and they don't know where else to get the information …so they just do it anyways and don’t realize the consequences. If my parents were able to talk to me about it, I would actually have more information instead of doing my own research or hearing stories from my friends”*.

(Female from Malaysia)

*“I wouldn't ask my mum to tell me about STIs she would just be like why do you need to know that information? Why don't you go to school? Maths is important, go and do maths. Sexual health is not a normal dialogue and it is not a normal thing to talk about”*.

(Female from Sudan)

*“…it is a Muslim country and it is difficult to talk about those things”*.

(Male from Brunei)

Participants from both groups agreed that compared to the conservative environments in their countries of origin, discussion of sexual health and HIV-related issues in WA appeared to be more acceptable.

*“… a lot of my friends when they come here are more open minded, everybody adapts to the culture so it is easier to talk about it”*.

(Female from Malaysia)

Social support from peers yielded a sense of belonging and was seen as a help-seeking enabler.

*“...when you have a sense of belonging you feel like you are part of the community … but once there is no social support and you feel lonely then you really don't feel like accessing anything”*.

(Male from Ghana)

### 3.3. Gender-Combined Information Sessions

Some SEA/EA participants referred to gender-combined education as a more conducive and informative environment for assimilating sexual health messages with a majority that preferred information sessions with both genders. While a fewer number of people acknowledged that combined sessions may be “awkward” and “embarrassing”, concluding that it should be voluntary rather than presumptive.

*“…we should take lessons together to get to know each other because we do not know what males think or females think so we have to take lessons together”*.

(Female from Japan)

“Sometimes we may feel embarrassed in groups. If we learn about the female body how will the men feel? and if we learn about the male body will the females feel?”

(Female from Taiwan)

The disparity in responses was an interesting finding that was not analysed in detail during the focus group sessions but was noteworthy and a point recommended for further investigation in future studies.

### 3.4. Privacy, Confidentiality, and Trust in Testing

The issue of confidentiality was a frequent response to the question on potential barriers for sub-Saharan African migrants. Concerns were mainly around the security of information, trusting the system, and repercussions of a positive HIV test, e.g., being exposed to investigation. However, based on the responses recounted by participants, privacy and confidentiality concerns may have been influenced by experiences in their countries of origin where healthcare professionals breached confidentiality by disclosing positive HIV results and consultation privacy was not assured.

*“… when you go into a clinic there is a section that says HIV area or there is a banner or a sign so anyone you see walking in there, everyone thinks they are HIV positive. There is no privacy”*.

(Male from Nigeria)

While participants had not experienced any confidentiality breach in Australia, they expected its occurrence as a result of past experience. A proposed solution was to incorporate sexual health services into general health care services, not isolating it as a stand-alone service.

*“…there should not be a special clinic for HIV, it should be like within a place where you can access a lot of services … like how we go into the supermarket and buy foodstuff it shouldn't be like a special corner”*.

(Female from the Gambia)

### 3.5. Tangible Limitations-Knowledge, Financial Constraints, and Language

There were substantial barriers found to reduce the likelihood of help-seeking even when health services were desired. Lack of knowledge about available sexual health services by the majority of SSA participants was attributed to the low frequency of sexual health messaging (for example no billboards or posters on HIV awareness) compared to other health issues like alcohol and drug use, which participants felt were the Australian Government’s foci.

*“…In terms of drugs, its everywhere people talk about it, there are a lot of adverts, the Government puts a lot more effort into it because they know that it impacts negatively on people, but when it comes to sex, we don't hear much about it”*.

(Male from Nigeria)

Financial constraints were barriers mentioned in relation to limitations in insurance cover and a general unaffordability of services among participants in both groups. To alleviate this problem, there were recommendations on better payment options, especially through instalments.

*“If I’m not able to afford treatment, then I can't afford to go”*.

(Female from Vietnam)

*“… consider the financial costs as well because we don't get access to medical assistance here like back home …”*.

(Female from Malaysia)

*“…the OSHC (Overseas student health cover) insurance, after you go for it, they say it is fine but a week later, you get a letter in the mail that you have to pay for pathology and it is expensive”*.

(Female from Namibia)

There was a general consensus among sub-Saharan African and Southeast Asian/East Asian participants on the sensitive nature of sexual health topics and participants felt that the inability to express themselves added complexity to sensitivity. Communication with someone from a similar background was found to ease help-seeking. Migrants who were proficient in English still cited persistent health literacy barriers in relation to understanding medical terminology.

*“…in terms of language, if you share a similar character or similar attitudes or beliefs it is very easy to access the services”*.

(Female from Malawi)

*“When we came here we spoke fine English but didn’t know medical terms.”*.

(Female from Sudan)

### 3.6. Knowledge Dissemination As a Stigma Reduction Strategy (Who, What, and How)

A few participants from the sub-Saharan African groups were able to highlight extended benefits of routine general check-ups in detecting other conditions, not just STIs. Both sub-Saharan African and Southeast Asian/East Asian participants considered knowledge about sexual health and existing services essential for making informed decisions. Most these sub-Saharan African participants were not exposed to sexual health education growing up, and for those that had received information the content was diluted and delivered mostly from a religious perspective. There were, however, exceptional, but rare, cases where sexual health education was taught comprehensively. This was quite contrary to experiences in the Southeast Asian/East Asian group as most participants were taught about sexual health in high schools with topics particularly focused on pregnancy and protection but also moderately exploring HIV and other STIs as part of the curriculum. Similar to the sub-Saharan African participants, there were no sexual health discussions in Southeast Asian/East Asian homes.

*“We learnt it specifically when we were 15 meaning we didn't know anything until we were 15…”*.

(Female from Malaysia)

*“…they teach us about sexual health from an early stage like high school from a religious point of view… it is mostly no sex before marriage and they kind of don’t encourage it. It is pretty much an abomination if you have it before marriage”*.

(Female from Zambia)

Participants were able to propose the “who”, “what”, and “how” perceived as requisites for sexual health education as seen in Table 3. Although some responses were similar between both groups (**), others were group-specific (* SEA/EA/* SSA—See Table 3 footnote).

### 3.7. Systemic Discrimination

Systemic discrimination refers to behaviour patterns, practices, or policies that are part of the structure of a system or organisation, which results in certain groups of individuals being disadvantaged [54]. Systemic discrimination may occur in different settings, two settings were cited by our participants, *i.e.*, an institutional and a social setting. Institutional systemic discrimination is described as institutions being biased in their dealings with members of racial minorities [55], this was experienced by a large number of participants in terms of employment, accommodation, and education. Social systemic discrimination refers to unequal treatment of an individual or a group of people by their counterparts by virtue of belonging to a social class or category perceived less favourably in a social environment [56]. Participants maintained that systemic discrimination was present particularly when seeking employment and accommodation where preference was almost always given to Australian permanent residents or citizens.

#### 3.7.1. Institutional Systemic Discrimination

##### Employment

Across both groups, participants expressed concerns about the limited opportunities for non-Australian permanent residents or citizens based on current practice. Current practice according to the Australian Department of Immigration and Border Protection is for Australian citizens and Australian permanent residents to be afforded preference for employment, following the priority system for recruitment and selection of candidates. Participants felt that applicants’ qualifications and experiences were not primary considerations in employment.

*“If you want to apply for a job like after you graduate or even internships they give offers mostly for Australians because they are residents…”*.

(Female from the Philippines)

*“Migrants may have better qualifications compared to mainstream Australians, the issue is systemic discrimination that is where the problem is, everything in Australia is all systemic, it is systemic racism and systemic discrimination. For example, if I apply for a job let us say with one of the departments, the issue will not openly be saying no to me, but it will be a systemic reason that will come through as to why I cannot get that job and someone else would end up getting it”*.

(Female from Zambia)

##### Language Barriers in Employment

Language was a factor specific to Southeast Asian/East Asian participants, who found that opportunities were limited because of their inability to communicate fluently in English.

*“…I went and talked to a manager and I said I am looking for job at the moment is there any position for me … he said oh because your English is not enough so I wouldn't hire you. That made me so sad ... he could have said no position at the moment, he shouldn't tell me that my English is not enough”*.

(Female from Japan)

##### Accommodation

Some sub-Saharan African participants had requests from homeowners to specify their countries of origin after which, on most occasions, they were denied residence. This led to concealing their identities and reiterating their legal rights to equal treatment.

*“… he asked where are you from? I said I am a student … he said which region of the world? I said I have the legal right to rent an apartment and I have the money so I do not understand. He said he is not racist…but I just knew immediately that it was purely discrimination because you don't have the right to ask for my region to be a condition for me to access opportunities”*.

(Female from Nigeria)

Conversely, almost none of the Southeast Asian/East Asian participants had problems securing accommodation and described the process as ‘easy’.

##### Educational Inequity

Southeast Asian/East Asian participants felt that education was equal and that it was determined by an individual’s ability to pay the fees.

*“…it is all about the money, as long as you can pay then you can go to Uni”*.

(Male from Indonesia)

A few participants from sub-Saharan Africa experienced racial discrimination with educational institutions in terms of services rendered. Reference was made to certain universities refusing to process students’ visas even with more years of study to completion, forcing these students to return home. A Zambian female who was the alleged first international student in an unnamed university to do an honours degree experienced unfairness in the allocation of supervisors.

*“…there were about 15 people in our class and every single one of them was given a supervisor by the university but I was told to look for my own supervisor, so I asked why that was and they said we have just ran out of supervisors”*.

(Female from Zambia)

#### 3.7.2. Social Systemic Discrimination

Social systemic discrimination refers to discrimination occurring in a social setting between people living with HIV and other members of their social circles. Within the SSA and SEA/EA groups, there were preconceived ideas about STI transmission and stereotypes of people living with HIV as promiscuous or immoral.

*“…when it comes to HIV they will think that person is more likely to get the disease from some kind of sexual experience or that person could be a drug addict so there will definitely be negative judgement…”*.

(Male from Ethiopia)

According to stories recounted by participants, people living with HIV experienced social or self-isolation mostly in their home countries or within their communities here in Australia.

*“When they have the STI, they don’t feel confident to communicate with anyone”*.

(Vietnamese female)

Words like ‘excommunicate’ and ‘shun’ were used to describe both societal and familial treatments of people living with HIV. These actions were rationalized by saying it was a protective mechanism to avoid being infected.

*“…it does not matter if you are brothers or sisters… when you are positive you are positive that means you are odd from the family, they don't care because you can still infect them…”*.

(Male from Burundi)

*“In my country if you have HIV people will not consider you as human anymore because many people are afraid of being infected”*.

(Male from Indonesia)

Almost all sub-Saharan African participants were appreciative of the exceptional knowledge levels on STIs in Australia. They felt that this allowed for a more conducive environment for people living with HIV and other STIs. Positive remarks were made towards the comprehensive support services provided in Australia compared to their countries of origin.

*“…support groups here have more resources and facilities. Over there it is mainly sitting under a tree and talking about how our life is over but over here there is a huge emphasis on your life isn't over, you can still go to school and you can still be a doctor or a teacher or whatever you want to do … because everyone else here is educated and you do not have to worry as much, it is much more accepting”*.

(Female from Sudan)

Nevertheless, some sub-Saharan African participants believed that not everyone was able to benefit from the favourable conditions in Australia as a result of stigma-related visa refusal for migrants living with HIV. Only one participant knew a person living with HIV that was able to successfully obtain a visa, other shared stories related to visa refusals. Participants expressed that the processes involved in appealing for visas for a person living with HIV were too tedious and caused most participants to nullify the visa application process.

*“Only a few cases would still come here even if they are HIV positive like one of the delegates who said it took her a whole process. It’s a frustrating process that would make many people just say no I am not going to go to Australia anymore because the process is too frustrating”*.

(Female from Zambia)

### 3.8. Health Service Disparities—Targeted Screening and Lack of Consent

Perceived attitudes of health care providers towards migrants in targeting them for STI screening were cited not only as a discriminatory experience but also a barrier to help-seeking. They felt that targeted screening engendered screening without consent. Participants expressed their desire to see healthcare workers treat all patients equally.

*“I think the people who start the stigmatization are the health professionals…some people are actually targeted like migrants and indigenous people as key carriers of HIV … I don't think I will like to go to a hospital where I may be a target so making it a universal test for everyone is better ”*.

(Male from Ghana)

## 4. Discussion

The results of this study extend and support previous studies on migrant sexual health by exploring barriers, enablers, and the underlying stigma and discrimination towards migrants and associated impacts on access to sexual health services. Our findings corroborate previous literature on the impact of the social determinants of health on sexual health help-seeking. While there were some common themes across both migrant groups, some were group-specific, illustrating the heterogeneity of people from culturally-diverse backgrounds [14]. Within each target group, themes were further subdivided to differentiate between those related to help-seeking barriers and enablers, and those associated with stigma and discrimination (Figure 1). Common barriers and enablers to help-seeking were sociocultural and religious influences, financial constraints, and knowledge dissemination to reduce stigma. Common experiences of stigma and discrimination in both groups were in terms of employment preference and the social and self-isolation of people living with HIV. Financial constraints, language, and experiences of stigma were barriers consistent with previous literature [37,57,58]. These barriers are further explored below.

### 4.1. Impact of Cultural and Religious Beliefs on Help-Seeking

Aspects of cultural and religious beliefs may influence the discussion and acceptance of sexual health communication strategies by migrants [17]. Sexual health continues to have a “taboo connotation” in migrants’ countries of origin, making it a topic that is rarely discussed.

Southeast Asian/East Asian participants frequently referred to their culture as “conservative”, a feature found to be both beneficial and detrimental to their sexual health [59]. While conservative behaviors may be protective factors for HIV and other STI transmission, a previous study had suggested that Asian migrants had low levels of knowledge on STIs, safe sex, and transmission mechanisms for HIV [59], establishing links between cultural influences and knowledge levels.

The Australian National Health and Medical Research Council (NHMRC) cultural competency framework is an approach to reorienting public health promotion programs to better meet the needs of CaLD communities [60]. This framework consists of four interrelating levels: systemic, organizational, professional, and individual, suggesting that cultural competence at the individual and professional level is underpinned by the capacity at the systemic and organizational level [60]. Cultural competence within the health system is best considered as the development of individual workers, agencies, and the health system, and not merely an awareness of existent cultural differences [61]. One direct recommendation from this study is to increase culturally-sensitive message delivery in order to disseminate information, allay suspicions, and establish trust between migrants and the health sector which will, in turn, increase service uptake. This may be done by actively engaging with migrant communities and relevant stakeholders in the development of sexual health messages. A good example can be seen in a resource developed by the metropolitan migrant resource center in partnership with the Western Australian AIDS Council. The resource ‘Your cultural lens’ is an online training program to facilitate cross cultural communication for sexual health and blood-borne viruses [3,62].

### 4.2. The Impact of Low Knowledge Levels

Our study found low levels of sexual health literacy amongst both sub-Saharan African and Southeast Asian/East Asian participants. Health literacy extends to knowing and understanding how to navigate the health care system [63]; however, nearly all of the migrants in our study were unaware of available sexual health services. Knowledge and education were highlighted as vital to create awareness of safer sexual practices, reduce stigma or as a tool to empower and protect migrant rights. Other studies involving sub-Saharan African and Southeast Asian/East Asian migrants have also reported that lack of knowledge increased the risk of contracting STIs [58,64,65,66]. An Australian study found that poor health literacy, navigating an unfamiliar health system, and making decisions from a different cultural perspective in a foreign language with obscure medical terminology used to explain health conditions were overwhelming experiences for migrants that needed to be considered by health care providers [58].

There were suggestions for education to be incorporated into school curricula or taught through media like workshops, seminars, drama, or events. The differences in the medium of message delivery serves as another example of diversity between migrant groups. Southeast Asian/East Asian participants preferred messages to be communicated through events delivered by ‘people of influence’. The success of this strategy may be supported by diffusion of innovations theory, which involves key opinion leaders and role models in the delivery of messages, and has been beneficial and successful in a number of HIV prevention interventions [67,68].

Sub-Saharan African participants suggested that the most appropriate medium would be drama, as it was tied to cultural learning methods in their countries of origin. Drama is an interactive art, popular for its influential role in actively engaging participants by creating auditory and visual expressions and facilitating discussions on relevant issues while drawing on participants’ life experiences [69,70]. Drama uses the ‘learned self-relevance’ process in message delivery, a process which enables participants to identify with characters in the drama or recreate their realities through role-play [70]. The benefits of drama as a strategy has been established in a number of countries worldwide and is increasing in popularity within the sexual health field because it enables understanding [69], is effective in increasing knowledge [71], is more culturally acceptable [69], and is reliable [72].

### 4.3. Past Experience and Stigmatization of People Living with HIV

Migrants’ perceptions and attitudes towards sexual health in their host countries were principally shaped by past experiences from their countries of origin, leading to an expectation of similar stigma levels in host countries. These experiences served and continued to serve as barriers to seeking help. For example, our study found an absence of social support for people with STIs in migrants’ home countries. Terms like *“outcast”, “excommunication”,* and *“not-human”* were used to describe attitudes towards people living with HIV who were seen as highly infectious. Our findings were consistent with a previous study conducted in Australia where migrants from resource-poor countries who had likened HIV to death and social ostracism based on past experiences, were more likely to delay help-seeking regardless of whether or not the stigma levels in the host country were similar to that of their countries of origin [27]. While breaches of confidentiality in healthcare settings predominantly occurred in their countries of origin, these experiences continued to shape perceptions and serve as barriers to help-seeking after migrating to Australia. This highlights a need to determine how expected stigma based on past experiences impacts on help-seeking. According to Christou [57], the ability of migrants to forge futures in an adopted country is a consequence of their past experiences and drawing on these backgrounds will inform a more creative capacity in service delivery that enables migrants to navigate constraints and barriers. Sexual health services need to be more focused on visibly demonstrating that these stigmatizing experiences are non-existent in Australia and portray a more welcoming approach during the promotion of sexual health services.

### 4.4. Stigmatization and Discrimination in Employment

Discriminatory and stigmatizing experiences have been found to exacerbate the risk of adverse health outcomes in CaLD communities [73,74,75]. Our study alongside others has identified experienced stigmatization as a significant barrier to help-seeking [63,76,77,78,79]. A large proportion of participants experienced one or more forms of stigmatization and discrimination in terms of obtaining accommodation, employment, healthcare, and education. Migration exacerbates the impacts of the social determinants of health and post-migratory stressors are further compounded by stigmatization which can have an indirect but strong effect on migrants’ attitudes and behaviours towards help-seeking [22,80]. Migrant health, therefore, extends beyond merely the management of sicknesses and diseases to an inherent link with the broader social determinants of health [22].

Particularly, discrimination in employment was common in both SSA and SEA/EA groups. The loss of pre-migration employment status was evident with a number of migrants transitioning into lower paying jobs despite previous professional expertise [81].

Migrants identified the role of systemic discrimination in employment citing a preference for Australian citizens or permanent residents over migrants for reasons including unrecognised credentials, lack of experience in Australia and language limitations. Previous literature has also identified similar reasons for employment preferences with additional factors including poor social networks, poor previous education quality and lack of information about the job market [82,83,84]. Another Australian study by Gibney *et al.* [85] concluded that unemployment was extensive among migrants, refugees, and asylum seekers despite high levels of relevant workplace skills.

Maslow’s Hierarchy of Needs suggests that individuals prioritize physiological needs over that of safety and health [86], and provides a framework to understand the basis for reduced willingness to access health care services when encountering unemployment. Unemployment results in financial constraints; this was common to both SSA and SEA/EA groups who reiterated that in the absence of finances, they would not seek help. Help-seeking may be diminished if health is not a priority, further complicated by perceived or actual financial loss associated with health care costs [27,81]. Neoliberal models of health, increasingly pervasive in high-income countries such as Australia [27], presume an autonomous, informed, and rational actor who weighs the cost and benefits to maximise gains, but when extrapolated to the migrant context, collectivist, and diverse backgrounds make health care decisions dependent on other financial, cultural, and social contexts [27]. Considerable collaborative and harmonized efforts from the Australian Government and migrant communities are required to tackle residency issues, language differences, accommodation difficulties, unemployment, and financial constraints [27]. Our study reinforces the need to work towards removing system, institutional, and regulatory barriers to receiving equal health care in migrants [3].

### 4.5. Stigmatization and Discrimination in Screening

Migrants from sub-Saharan Africa experienced discrimination as a result of targeted STI screening. They felt deprived of their rights to informed consent, which made them hesitant to seek help. Undoubtedly, targeting priority populations or high-risk groups by identifying and distinguishing them from other low-risk groups enables the targeted allocation of resources and provision of specific sexual health interventions and services [87,88]. This approach successively instigates a protective benefit on the entire community. However, according to the 2011 Australian National HIV Testing Policy [89], even in situations deemed to be beneficial for public health, testing for STIs must remain voluntary and based on informed consent without coercion or compulsion. The policy uses the term “informed consent” to stress the importance of health care providers providing sufficient information that may or may not facilitate testing [90].

These procedures were not experienced by some migrants in our study with reports of blood-sample collection with no clear indication on what was being tested, after which testing was carried out without their permission or informed consent. There is legislation in place to protect migrants from testing without consent; however, migrants in our study were unaware of their rights, hence their request for more education that focuses on important messages about the Australian law regarding sex, HIV, and immigration. Participants indicated a desire to understand their rights to enable a sense of security in their new environment, and to know what can be challenged in the face of discrimination.

## 5. Conclusions

Although challenging, it is important to separate migrant populations to whatever degree that is possible in order to have a better understanding of their sexual health needs. We acknowledge that several sub-communities and sub-classifications exist within the migrant population with some individuals identifying with more than one group. However, our study has shown that there is a reduced likelihood of success in universal migrant health service delivery. Furthermore, within-region fragmentation of migrant communities may then be the subject of future studies for more transferable findings. In order to overcome the barriers to sexual health testing and increase service uptake, there needs to be routine dissemination of culturally-appropriate knowledge and information specific to the needs of targeted groups. Stigmatization and discrimination may be linked to pre-existing prejudice and past experience making it difficult to reduce overall. However, curtailing stigmatization and discrimination in healthcare settings will be beneficial in enabling early help-seeking among migrants. Migrants require knowledge of the host country’s laws and their individual rights as an empowerment tool to challenge discrimination and make informed decisions commensurate with actual rather than perceived knowledge. Overcoming barriers, imparting sexual health knowledge, and reducing stigmatization and discrimination where possible will simultaneously accelerate help-seeking and result in better sexual health outcomes in priority populations.

Our qualitative study created a platform for the voice of SSA and SEA/EA migrants and their experiences to be heard, which may otherwise remain overlooked and undiscovered by studies that homogenize migrants as one body. We report first-hand evidence, usable by sexual health service providers and policy makers to better understand the reasons behind migrants’ help-seeking behaviours. Translating these findings into practice and policy, whilst a challenging prospect, will address both migrant sexual health barriers and social inequities and bring about substantial benefits that will outweigh the eventual costs. The findings have implications for policy in relation to better informed decision-making and resource allocation that facilitates migrant help-seeking and access to health services.

## Figures and Tables

**Table 1:** Focus group discussion domains and sample questions.

Domain	Sample Question
Experience of stigma and discrimination as migrants	*How do opportunities differ between migrants and non-migrants in terms of accessing health services?*
Cultural influences on sexual health education	*Tell me a story about sexual health education that you received growing up in your home country.*
Help seeking—accessing health services	*Where can you go for a sexual health check-up?*
Experience of stigma and discrimination related to sexual health	*Often Sexually Transmitted Infections (STIs) are linked with shame/embarrassment, why do you think this is the case?*
Identifying barriers and enablers of sexual health	*What would encourage people to access sexual health services?*
Appropriate sexual health messaging	*Which sexual health topic is the most relevant for your community and why?*
Identifying best approach to delivering messages	*What are your ideas on the best ways to communicate sexual health messages?*

**Table 2:** Demographics of study participants.

Characteristics	Number of Participants				
	Sub-Saharan Africa	Southeast Asia	East Asia	Other Regions	All Participants
Gender					
Male	10	3	1	3	17
Female	11	8	7	2	28
Age group (years)					
18–28	14	8	0	2	24
29–39	5	2	7	3	17
40–50	2	1	1	0	4
Religion					
Christian	19	6	1	1	27
Muslim	2	1	0	4	7
Buddhism	0	3	1	0	4
Not religious	0	1	6	0	7
Years Lived in Australia					
1–5	14	9	6	5	34
6–10	4	2	2	0	8
>10	3	0	0	0	3
Education Level					
Primary School	1	0	0	2	3
Year 12	1	4	3	0	8
TAFE/Diploma	2	3	4	1	10
University bachelor degree or higher	17	4	1	2	24

**TAFE**—Technical and Further Education qualification.

**Table 3:** Knowledge dissemination recommendations from participants.

Question	Participants’ Response	Theme and Representative Quote
Who? Groups to target	Target the young (**)	Retainment *“…teaching kids at a young age would be very useful… when they are young then they have something to follow…they will listen to you, but if you keep them in the dark then that makes them want to discover on their own which is more dangerous”* (Male from Laos) Empowerment *“...when you are young you obviously want to do something when you grow up and you are passionate…”* Male from Burundi)
What? Priority messages for communication	Australian law regarding sex, HIV and immigration (**)	Feeling secure *“…you should know you have a right and it is protected by law and if something is against you then you know what to do”* (Male from Ghana)
	Sexually transmitted diseases (* SSA)	False speculation *“…most people think that there is no HIV in Australia and when they get here they just let go”* (Female from Zambia)
How? Acceptable mediums for communicating sexual health	Drama (* SSA)	Culturally similar approach *“…we are naturally story tellers and that is how our parents used to explain things to us don't do this don't do that using exaggerated elaborate stories and that is the way we learn things…”*(Female from Sudan)
	Peer education (**)	Easier adoption of messages *“…you can organise a group so everybody can know about sex and then go to their peers, I will feel more comfortable talking about sex with my peer than an older woman like 50 years old…”* (Female from Liberia)
	Events (* SEA/EA)	Trust in credentials *“At home…we have events so they get highly trained professionals and people who have credentials because that is who people trust …if some random person just comes up and tries to get information across it is not going to sink in unless someone who has credentials… like a professor in health science or mental health…”* (Male from Laos)
	Adverts and commercials (* SEA/EA)	Broad coverage and engaging *“…Commercials being shown every day or every couple of hours, like on the TV or in universities …because at this age group we are attracted to bright visuals and it will speak to us…”* (Female from Malaysia) *“Through the media because everyone watches it, it can be on videos to pass message across and you can reach a lot of people”* (Female from Vietnam)

Notes: (**)—Common response from Southeast Asian/East Asian and sub-Saharan African participants; (* SEA/EA)—Response specific to the Southeast Asian/East Asian group; (* SSA)—Response specific to the sub-Saharan African group.
